# Estimated Monetary Value of Future Research Clarifying Uncertainties Around the Optimal Adult Hearing Screening Schedule

**DOI:** 10.1001/jamahealthforum.2022.4065

**Published:** 2022-11-11

**Authors:** Ethan D. Borre, Evan R. Myers, Judy R. Dubno, Susan D. Emmett, Juliessa M. Pavon, Howard W. Francis, Osondu Ogbuoji, Gillian D. Sanders Schmidler

**Affiliations:** 1 Department of Population Health Sciences, Duke University School of Medicine, Durham, North Carolina; 2 Duke-Margolis Center for Health Policy, Duke University, Durham, North Carolina; 3 Division of Women’s Community and Population Health, Department of Obstetrics & Gynecology, Duke University School of Medicine, Durham, North Carolina; 4 Department of Otolaryngology–Head and Neck Surgery, Medical University of South Carolina, Charleston, South Carolina; 5 Department of Head and Neck Surgery and Communication Sciences, Duke University School of Medicine, Durham, North Carolina; 6 Duke Global Health Institute, Duke University, Durham, North Carolina; 7 Division of Geriatrics, Department of Medicine, Duke University School of Medicine, Durham, North Carolina; 8 Center for Policy Impact in Global Health, Duke Global Health Institute, Durham, North Carolina; 9 Duke Clinical Research Institute, Duke University School of Medicine, Durham, North Carolina

## Abstract

**Importance:**

Adult hearing screening is not routinely performed, and most individuals with hearing loss (HL) have never had their hearing tested as adults.

**Objective:**

To project the monetary value of future research clarifying uncertainties around the optimal adult hearing screening schedule.

**Design, Setting, and Participants:**

In this economic evaluation, a validated decision model of HL (DeciBHAL-US: Decision model of the Burden of Hearing loss Across the Lifespan) was used to simulate current detection and treatment of HL vs hearing screening schedules. Key model inputs included HL incidence (0.06%-10.42%/y), hearing aid uptake (0.54%-8.14%/y), screening effectiveness (1.62 × hearing aid uptake), utility benefits of hearing aids (+0.11), and hearing aid device costs ($3690). Distributions to model parameters for probabilistic uncertainty analysis were assigned. The expected value of perfect information (EVPI) and expected value of partial perfect information (EVPPI) using a willingness to pay of $100 000 per quality-adjusted life-year (QALY) was estimated. The EVPI and EVPPI estimate the upper bound of the dollar value of future research. This study was based on 40-year-old persons over their remaining lifetimes in a US primary care setting.

**Exposures:**

Screening schedules beginning at ages 45, 55, 65, and 75 years, and frequencies of every 1 or 5 years.

**Main Outcomes and Measures:**

The main outcomes were QALYs and costs (2020 US dollars) from a health system perspective.

**Results:**

The average incremental cost-effectiveness ratio for yearly screening beginning at ages 55 to 75 years ranged from $39 200 to $80 200/QALY. Yearly screening beginning at age 55 years was the optimal screening schedule in 38% of probabilistic uncertainty analysis simulations. The population EVPI, or value of reducing all uncertainty, was $8.2 to $12.6 billion varying with willingness to pay and the EVPPI, or value of reducing all screening effectiveness uncertainty, was $2.4 billion.

**Conclusions and Relevance:**

In this economic evaluation of US adult hearing screening, large uncertainty around the optimal adult hearing screening schedule was identified. Future research on hearing screening has a high potential value so is likely justified.

## Key Points

**Question:**

What is the monetary value of future research on adult hearing screening in the US?

**Findings:**

In this model-based economic evaluation, the value of research clarifying all uncertainties around the optimal adult hearing screening schedule was found to be between $8.2 and $12.6 billion.

**Meaning:**

Investing up to $8.2 to $12.6 billion in a research portfolio on adult hearing screening in the US is likely a good use of resources.

## Teaser

This economic evaluation projects the monetary value of future research clarifying uncertainties around the optimal adult hearing screening schedule.

## Introduction

Hearing loss (HL) is the fourth leading cause of disability worldwide and more than 50% of US adults aged 70 years and older have HL.^1,2^ While effective treatments for HL are available, most US adults do not have their hearing tested and are unable to access these treatments. Hearing screening programs increase HL diagnosis and treatment uptake.^3,4,5,6^ However, there are uncertainties around the optimal age of screening initiation, the generalizability of trial outcomes beyond predominately veteran populations, and the lack of randomized clinical trial evidence linking hearing screening to downstream quality of life, communication, and general health improvements.^7^ Results from future research studies may reduce the hearing screening health policy decision uncertainty, but their contributions must be weighed against the costs of conducting such studies.

Value of information analysis (VOI) is a method to project the expected monetary value of a research project given its contributions to a decision problem beyond current knowledge, including whether the new information would change actions.^8^ The VOI estimates the reduction in the probability of making a wrong decision after completion of the research project (and reduction of decision uncertainty), multiplied by the financial and health consequences of that wrong decision.^8,9^ This value can then be compared with the projected cost of a research project to project the upper dollar amount that should be allocated to a research project at a given societal willingness-to-pay (WTP) threshold.

This study’s objective was to estimate the potential economic value of future research reducing uncertainties in the evidence around adult hearing screening in the US. We extended a model-based cost-effectiveness analysis of adult hearing screening programs in the US to perform VOI.^10,11^ Decision makers can use these results, compared with the projected cost of a research study, to make investment decisions and maximize patient welfare.

## Methods

### Analytic Overview

For this economic evaluation, we used the previously validated Decision model of the Burden of Hearing loss Across the Lifespan (DeciBHAL-US), parameterized with model inputs from a recent cost-effectiveness analysis of adult hearing screening in the US.^10,11^ We first assigned distributions to uncertain model inputs from the prior analysis to perform probabilistic uncertainty analysis (PUA). We then calculated the expected value of perfect information (EVPI) and expected value of partial perfect information (EVPPI) by projecting decision uncertainty reductions associated with reductions in model input uncertainty. We followed the Professional Society for Health Economics and Outcomes Research (ISPOR) best practices for VOI and the Second Panel on Cost-Effectiveness in Health and Medicine and adhered to the Consolidated Health Economic Evaluation Reporting Standards (CHEERS) reporting guideline.^12,13,14^ We used a cost-effectiveness acceptability curve and net monetary benefits to see how WTP was associated with the certainty about the optimal screening schedule (base case WTP = $100 000/quality-adjusted life-year [QALY]).^15,16^ This study was exempt from institutional review board review because it did not involve human participants.

### Model Description

DeciBHAL-US is a microsimulation model of HL natural history, diagnosis, and treatment primarily based on National Health and Nutrition Examination Survey epidemiologic data.^10^ Simulated male patients and female patients experience yearly age-specific probabilities of acquiring HL, either sensorineural HL or conductive HL, worsening of their HL, and receiving or discontinuing treatment (Table 1).^2,3,6,7,11,17,18,19,20,21,22,23,24,25,26,27,28,29,30,31,32,33,34,35^ We included hearing aids as treatments for persons with sensorineural and conductive HL (defined as better ear pure tone average [PTA] of 25 dB HL) and cochlear implants as treatments for persons with severe and profound sensorineural HL (defined as better ear PTA of 60 dB HL). DeciBHAL-US collects patient outcomes in terms of QALYs, based on HL severity and treatment status, and costs in 2020 US dollars from a health systems perspective.

**Table 1.** Selected Model Inputs

Clinical input parameters	Value		Distribution	Mean (95% CI)	Reference
	Males	Females			
**Bilateral SNHL probability, yearly, %**					
Age, y					
40-45	0.76	0.06	NA	NA	Goman and Lin,^2^ 2016; Homans et al,^17^ 2017; Cruickshanks et al,^18^ 1998; Van Naarden et al,^19^ 1999
46-55	1.22	0.36	NA	NA	
56-65	2.33	1.25	NA	NA	
66-75	5.39	3.83	NA	NA	
≥76	10.42	9.17	NA	NA	
**SNHL progression, PTA decline in dB**	**Mean (SD)**				Lee et al,^20^ 2005
Ages 35-64 y	1.05 (0.4)		NA	NA	
Ages ≥65, PTA <40 dB HL	1.37 (0.4)		NA	NA	
**Yearly probability of HA uptake, %** ^a^	**PTA<40 dB HL**	**PTA ≥40 dB HL**			
Age, y					
40-55	0.54	2.35	NA	NA	Chien and Lin,^21^2012; Simpson et al,^22^ 2019
65	0.51	4.60	NA	NA	
75	0.60	8.14	NA	NA	
85	0.71	7.20	NA	NA	
**Yearly probability of HA discontinuation, ages 18+, %** ^a^	**Value**				
1 y After use	12.90		NA	NA	Takahashi et al,^23^ 2007; Kochkin,^24^ 2010
≥10 y After use	3.50		NA	NA	
**Yearly probability of cochlear implantation, %**					
Adults with severe or profound hearing loss with HAs, %	1.32		NA	NA	American Cochlear Implant Alliance^25^
**Health state utility values**					
Hearing loss					
None	0.84		NA	NA	Kaur et al,^26^ 2020; Grutters et al,^27^ 2007; Salomon et al,^28^ 2015
Mild (PTA 25-34 dB HL)	0.71		NA	NA	
Mild-moderate	0.68		NA	NA	
Moderate	0.65		NA	NA	
Moderate-severe	0.58		NA	NA	
Severe	0.54		NA	NA	
Profound	0.53		NA	NA	
Utility benefit of HAs	+0.11		Beta	0.11 (0.07-0.14)	Kaur et al,^26^ 2020; Grutters et al,^27^ 2007; Davis,^29^ 2007; Kaalund et al,^30^ 2021
Utility benefit of cochlear implants	+0.16		NA	NA	Kaalund et al,^30^ 2021
Screening effectiveness, multiplier on HA uptake	1.62		Normal	1.62 (1.12-1.93)	Yueh et al,^3^ 2010; Zazove et al,^6^ 2020; Borre et al,^11^ 2022
Screening test false positive probability, %	24		Beta	0.76 (0.68-0.83)	US Preventive Services Task Force,^7^ 2021
**Economic input parameters**	**Value, 2020 USD**				
Screening test cost	2		NA	NA	Liu et al,^31^ 2011
Audiology diagnostic test cost	295		Gamma	295 (8-1090)	Hojjat et al,^32^ 2017
Hearing aid device(s) cost	3690		Gamma	3690 (100-14 350)	National Academies of Sciences, Engineering, and Medicine,^33^ 2016
Yearly HA recurring cost	910		Varied along with device cost	NA	Hojjat et al,^32^ 2017; National Academies of Sciences, Engineering, and Medicine,^33^ 2016
Cochlear implantation cost	54 380		NA	NA	Hojjat et al,^32^ 2017; Semenov et al,^34^ 2013; Wyatt et al,^35^1996
Yearly recurring costs, cochlear implantation	1260-1400		NA	NA	

Abbreviations: HA, hearing aid; HL, hearing level; NA, not applicable; PTA, pure tone average; SNHL, sensorineural hearing loss.

^a^ Linear interpolation was used between ages not displayed.

### Simulated Screening Schedules

We simulated screening schedules that varied in age at initiation, ages 45, 55, 65, and 75 years, and frequency (either every 1 or 5 years; a total of 8 schedules tested). These ages and frequencies were selected in collaboration with HL and geriatrics clinical and policy experts. The effects of simulated hearing screening were incorporated as a multiplier on hearing aid uptake during the year of screening.

### Probabilistic Uncertainty Analysis

We assigned distributions to 5 uncertain model parameters identified as most influential in deterministic sensitivity analysis (utility benefit of hearing aids, screening effectiveness, screening test false positive probability, audiology diagnostic test cost, and hearing aid device cost; Table 1).^2,3,6,7,11,17,18,19,20,21,22,23,24,25,26,27,28,29,30,31,32,33,34,35^ We then ran 1000 iterations of the simulation, each iteration drawing from specified distributions.^36^ In this way, we estimated the simultaneous effect of uncertainty for these 5 parameters on the results. Simulating all potential screening schedules and comparing incrementally, we compiled the incremental cost-effectiveness ratios (ICERs) from each iteration. We generated a cost-effectiveness acceptability curve (CEAC) by comparing each screening schedule’s ICER with varying WTP values and determining the optimal schedule for that simulation iteration. The optimal schedule was defined as the most effective nondominated schedule that fell below the defined WTP.

### Value of Information Analysis

We followed previously published guidelines and methods to conduct the VOI analysis.^8,12,37^ To project the EVPI, we calculated the net marginal benefit of each schedule for every iteration of the PUA. The net monetary benefit is defined as the product of the expected effectiveness (QALYs) and WTP, minus the expected costs. The optimal schedule is identified under each PUA iteration as the schedule with the highest positive net monetary benefit. We compared the net monetary benefit of the optimal strategy under each PUA with that of the expected optimal schedule (produced using the mean of each given distribution). The sum of the differences between the maximum net marginal benefit of each PUA iteration (decision with perfect information) and the expected average net monetary benefit is equivalent to the EVPI. Projected to the population level, the EVPI represents an estimated upper bound on the dollar amount of justifiable research investments.

To estimate EVPPI for hearing screening effectiveness (defined in this study as a risk ratio for hearing aid uptake), we ran outer and inner loop simulations. Unlike EVPI, EVPPI is focused on a single model parameter as opposed to all model parameters. In the inner loop simulation, DeciBHAL-US drew from 4 parameter distributions, analogous to the PUA. For each PUA inner loop iteration, DeciBHAL-US drew from the defined screening effectiveness distribution as the outer loop. This allowed for the computation of the difference between the net monetary benefit of each outer loop iteration compared with the expected net monetary benefit to calculate the EVPPI in a similar manner to EVPI. The resulting EVPPI is interpreted as the maximum monetary value of acquiring perfect information around the effectiveness of hearing screening—or the upper bound of the cost of research studies (including clinical trials) on US adult hearing screening. This research portfolio would need to inform the risk ratio of hearing aid uptake among those screened for HL compared with current practice. In this study’s simulation, we ran 200 iterations of the inner loop and 50 iterations of the outer loop. To calculate the population EVPI and EVPPI, we multiplied the per-person EVPI and EVPPI by the estimated number of people ages 40 years and older in the US who currently have bilateral HL and are not treated (prevalent population) and the number of people expected to acquire HL over the next 5 years (incident population; incorporating a 3% discount factor).^2,12,21,38^

### Clinical Input Parameters

Simulated persons began at age 40 without HL and experienced yearly probabilities of HL (Table 1).^2,3,6,7,11,17,18,19,20,21,22,23,24,25,26,27,28,29,30,31,32,33,34,35,39,40,41,42,43,44,45,46^ Worsening of sensorineural HL was incorporated as a yearly dB increase in PTA (mean [SD], 1.05 [0.4] dB/y).^20^ After acquiring HL of any type, simulated persons have age-specific and severity-specific probabilities of HL diagnosis and hearing aid uptake (0.5%-8%).^21,22^ Hearing aid discontinuation varies along with the time since acquisition (13% in year 1 to 4% in year 10).^23,24^ Hearing aid users with severe and profound HL have a 1.3% yearly probability of cochlear implantation.^25,47^ The utility benefits of hearing aids (+0.11, beta distribution; 95% CI, 0.07-0.14) and cochlear implants (+0.16) were from a recent systematic review.^26,28,29^

We included hearing screening effectiveness as the inverse variance weighted average of 2 trials of hearing screening using a sound hearing test (1.62, normal distribution).^3,6,11^ While the SAI-WHAT trial only included patients with screening detected greater than 40 dB HL,^3^ we applied the screening effectiveness parameter to persons with any HL (≥25 dB HL) and varied the screening effectiveness parameter across its distribution. Additionally, we assumed that the risk ratio for seeking hearing health care was equivalent to that of hearing aid uptake in 1 study that only measured seeking hearing health care. This combined screening effectiveness parameter was synthesized for the purposes of VOI but given the differences in design between the 2 studies it should not be used as a stand-alone estimate of the effectiveness of hearing screening. We used a false positive probability (1−specificity) of 24% to account for imperfect implementation, which was used to calculate the costs of screening and assigned a beta distribution.^7^

### Economic Input Parameters

The costs of HL diagnosis and treatment included audiology diagnostic testing ($295) and 2 hearing aid devices ($3690).^32,33^ Both parameters were assigned gamma distributions, assuming variance equal to the mean as is standard in PUA when variance is not reported for cost parameters.^37^ Recurring costs included 5-yearly hearing aid device replacement and battery costs. Cochlear implant costs were $53 380 for implantation, with recurring costs of $1240 to $1400/y.^32,34,35^ The costs of screening were applied to persons without and with HL ($2/sound screening test).^31^ For persons without HL, 43% of persons receiving a false positive screen acquired a diagnostic test.^6,7,31,32^ For persons with HL, 51% of persons receiving a true positive screen were assumed to pursue an audiology diagnostic test but not acquire hearing aids.^6,31,32^

## Results

### Expected Cost-effectiveness Results

The expected discounted lifetime QALYs for current detection (CD) was 18.11 and ranged from 18.12 to 18.13 QALYs for the yearly screening schedules. Expected discounted lifetime costs were $1180 for CD and ranged from $1580 to $2480 for the yearly screening schedule. Yearly screening beginning at ages 75, 65, and 55 years all were cost-effective, with ICERs of $39 200 per QALY, $45 600 per QALY, and $80 200 per QALY. The ICERs for yearly screening beginning at age 45 years was $231 700 per QALY.

### Probabilistic Uncertainty Analysis

The PUA results showed that the optimal schedule was highly uncertain; there was no schedule that was optimal in greater than 40% of simulations at any WTP between $50 000 and $100 000 per QALY. At a base case WTP of $100 000 per QALY, yearly screening beginning at age 55 years was the optimal schedule in 38% of the simulations (Figure). Yearly screening beginning at age 45 years was optimal in 30% of simulations, yearly screening beginning at age 65 in 16%, CD in 11%, and yearly screening beginning at age 75 years in 6%. At lower WTPs, assuming payers are unwilling to pay more than $50 000 per QALY, CD was more frequently the optimal schedule. At WTPs higher than $100 000 per QALY, yearly screening beginning at age 45 years was frequently the optimal schedule (Figure).

**Figure.**
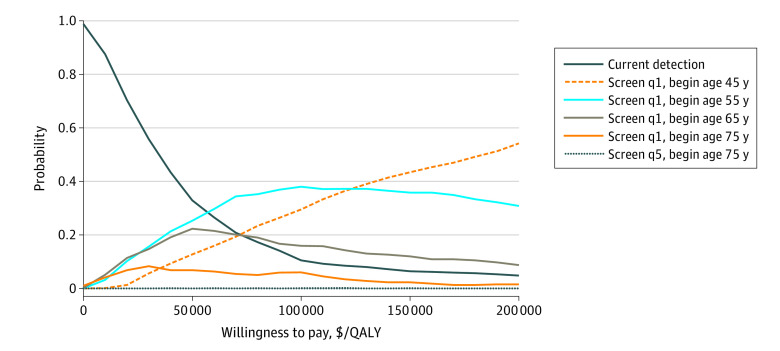
Cost-effectiveness Acceptability Curve The Figure depicts the probability that each simulated screening schedule is the optimal schedule (ie, the nondominated schedule with the highest effectiveness and incremental cost-effectiveness ratio under the willingness to pay) on the y-axis, across willingness to pay values ranging from $0 to $200 000 per QALY on the x-axis. QALY indicates quality-adjusted life-year; q1, yearly screening; and q5, screening every 5 years.

### Expected Value of Perfect Information

At WTP of $100 000 per QALY, the per-person value of reducing all uncertainty in the hearing screening decision problem was $176 (Table 2). Extending this value to all US adults aged 40 years and older with untreated HL, as well as those expected to acquire HL over the next 5 years, the population EVPI was $9.6 billion. Varying WTP assumptions, the population EVPI ranged from $8.2 to $12.6 billion.

**Table 2.** Expected Value of Perfect Information and Expected Value of Partial Perfect Information for Screening Effectiveness Across Varying Willingness to Pay Thresholds^a^

Willingness to pay, $/QALY	Perfect information		Partial perfect information	
	Expected value, $	Population expected value (billions)	Expected value, $	Population expected value (billions)
50 000	234	12.641	23	1.270
100 000	176	9.555	45	2.434
150 000	152	8.244	38	2.038
200 000	167	9.020	58	3.148

Abbreviation: QALY, quality-adjusted life-year.

^a^ All costs are presented in 2020 US dollars.

### Expected Value of Partial Perfect Information

The value of reducing all uncertainty on the hearing screening effectiveness parameter was projected using EVPPI analysis. At the base case WTP of $100 000 per QALY, the per-person EVPPI for screening effectiveness was $45 (Table 2). Extended to the current and future population affected by this decision over the next 5 years, the population EVPPI was $2.4 billion. This result suggests spending up to $2.4 billion on a hearing screening research portfolio that clarifies the effectiveness of hearing screening would be a cost-effective investment.

## Discussion

In this economic evaluation, the stochastic modeling analysis demonstrated large uncertainties in the optimal hearing screening schedule for US adults. These results expand the recent US Preventive Services Task Force evidence review on hearing screening by assigning a monetary value to uncertainties outlined within the report (such as quality of life benefits of hearing screening), as well as other economic uncertainties.^7^ At the US standard WTP, yearly screening beginning at age 55 years was most frequently the optimal schedule, although initiation at ages 45 and 65 years and no screening (CD) were each the optimal schedules in more than 10% of simulations. This high level of decision uncertainty due to imperfect current information, combined with the substantial prevalence of untreated HL, led to large EVPI estimates that ranged from $8.2 to $12.6 billion. Comparing this study’s EVPI estimate with published EVPIs of other health topics included in a 2016 systematic review, this study’s EVPI for hearing screening was higher than 83 of 86 other health topic estimates.^48^ Eliminating the uncertainty around the effectiveness of hearing screening alone, the population EVPPI was $2.4 billion, and we project investing up to $2.4 billion in research efforts that clarify the effectiveness of hearing screening to be a good use of resources.

Comparing this study’s estimated EVPPI of a hearing screening research portfolio with costs of recently completed trials in hearing health care, there is room for further research to be conducted. For instance, the NIH-reported costs of the Aging, Cognition, and Hearing Evaluation in Elders (ACHIEVE) randomized clinical trial totaled $16.5 million.^49^ This trial seeks to evaluate the effect of hearing aid intervention vs aging education control on incident dementia and is recruiting 850 patients with a 3-year follow-up.^50^ Clinical trials of hearing screening that help inform the risk ratio of hearing aid uptake compared with CD might be conducted over an even shorter timeline, and for much less than the EVPPI estimate of $2.4 billion. Additionally, this study’s EVPPI focused on screening effectiveness but, ideally, a future study would not define hearing aid uptake (alone) as the main outcome but would include measures of quality of life, function, etc, to clarify more uncertainties. Over time, as uncertainty is reduced or increased with new research, the EVPI and EVPPI will likely change proportionally with the amount of uncertainty remaining.

Further, this study’s total EVPI suggests that research around hearing health care may be underfunded in the US. The National Institute on Deafness and Communication Disorders 2021 budget awarded to hearing research was $212 million, substantially lower than the adult hearing screening research value of $8.2 to $12.6 billion.^51^ While this study’s EVPI and EVPPI estimates were over a 5-year time horizon, the timeline of resource investment would depend both on available funds and competing research areas.^52^ Nevertheless, given the large clinical and economic consequences of adult hearing screening, investing funding and research efforts into identifying the optimal schedule is warranted. With the recent US Food and Drug Administration release of over-the-counter hearing aids regulation,^53^ future research is needed to identify the effects of decreased hearing aid cost—and potential increased hearing aid use—on clinical outcomes and VOI estimates.

While these results give an upper bound on the monetary value of perfect and partial perfect information for adult hearing screening, they cannot estimate the value of a particular study or study design. Extended VOI methods, such as the expected value of sample information (EVSI), might be implemented in future research.^54^ Further, EVSI can inform trial design and sample size calculation and account for imperfect implementation to better inform funding agencies about which research studies to fund.^55^ Although computational power has been a limiting factor in calculating EVSI, novel methods might allow for more efficient estimation in future research studies.^56^ That said, a study that randomizes (or assigns similar groups of) adults to screening vs no screening and measures the proportion accurately identified by screening, uptake of hearing aids in those with confirmed HL, and benefit in terms of health outcomes over a meaningful duration should be considered.

### Limitations

This study’s analysis is strong in that, to our knowledge, it is the first analysis to incorporate the best available evidence to estimate of the value of future research in US adult hearing screening. However, this study has several limitations. First, the model included simplified assumptions around HL natural history, treatment uptake, and screening implementation. We detailed and justified these assumptions here and elsewhere and whenever possible chose to be conservative (ie, lower EVPI) in the assumptions.^10^ Second, given computational limitations, we did not assign distributions to every model parameter. Instead, we identified the most influential parameters in deterministic analysis, as well as the most uncertain parameters from a clinical and policy perspective, to explore in PUA. Third, we varied the false positive probability across its plausible range but did not simultaneously vary screening sensitivity. This was because we did not explicitly incorporate sensitivity in the study’s calculations, but rather included screening effectiveness as downstream hearing aid uptake. Future analyses might better explore the sensitivity and specificity of specific hearing screening modalities. Fourth, VOI methods only allow for estimation of the upper bound of monetary value of a research portfolio and do not suggest an amount that should be invested (as research projects in reality will not eliminate all uncertainties). However, EVPI and EVPPI can be compared with the costs of research portfolios to aid decision makers in understanding what is more or less likely to represent a good investment. Fifth, due to uncertainty in the evidence, we did not incorporate effects of HL treatment on dementia, but to the extent that HL treatment reduces the incidence and/or severity of dementia, the EVPI would increase given the large clinical and economic effect of dementia on this population. Lastly, we only estimated EVPPI for the screening effectiveness parameter. We selected this parameter for EVPPI analysis based on expert opinion on the most uncertain parameter in the general population and most substantial research gap.^7^ Future research might project the EVPPI for other model parameters, which would estimate the value of research portfolios clarifying that uncertainty but would not change the present study’s estimated screening effectiveness EVPPI.

## Conclusions

In this economic evaluation, the model-based analysis projects high potential value of future research on adult hearing screening, and in particular the effectiveness of hearing screening on increased hearing aid uptake. The high degree of uncertainty around the optimal age of hearing screening initiation and frequency, paired with the substantial burden of HL in the US, justifies research investment to identify the best hearing screening decision for millions of US adults with HL.
